# Lived experiences of migrant and refugee parents: Challenges encountered during their journey and settlement in Europe

**DOI:** 10.1016/j.jmh.2024.100294

**Published:** 2024-12-24

**Authors:** Elena Rousou, Paraskevi Apostolara, Venetia Sofia Velonaki, Irena Papadopoulos, Athena Kalokairinou, Ourania Sakellaraki, Victor Dudau, Andrea Kuckert, Runa Lazzarino, Manuela Mauceri, Alfonso Pezzella, Christiana Kouta, Theologia Tsitsi

**Affiliations:** a Assistant Professor, Department of Nursing, Cyprus University of Technology, 15 Vragadinou str, 3041, Limassol, Cyprus; b Assistant Professor, Department of Nursing, Egaleo Park Campus, University of West Attica Scientific Collaborator, National and Kapodistrian University of Athens, Ag. Spyridonos Str., Egaleo, 12243, Athens, Greece; c Assistant Professor, Department of Nursing, National and Kapodistrian University of Athens, 123 Papadiamantopoulou str, 115 27, Athens, Greece; d Professor of Transcultural Health and Nursing, Department of Mental Health and Social Work, Middlesex University UK, The Burroughs, London NW4 4BT, UK; e Professor in Community Nursing, Director of Laboratory Community Nursing, Faculty of Nursing, National and Kapodistrian University of Athens, Greece; f Former Project Manager, Former Consultant for MDM Greece, 12 Sapfous str, 10553 Athens, Greece; g Trainer, Edunet Organization, Basarabia E2 ap 19 200056 Craiova, Romania; h Lecturer Bachelor of Science in Health Studies, Vorarlberg University of Applied Sciences, CAMPUS V, Hochschulstraße 1, 6850 Dornbirn, Austria; i Research Assistant, Research Centre for Transcultural Studies in Health, Middlesex University, School of Health and Education, Middlesex University UK, The Burroughs, London NW4 4BT, UK; j Training tutor, C&B Formazione Professionale, Via Gorizia 36c, 95129, Catania, IT; k Temporary Professor, Department of Medical and Surgical Sciences and Advanced Technologies, University of Catania, Piazza Università, Catania, IT; l Lecturer in Mental Health, Director of Programmes and Senior Lecturer in Mental Health, Research Centre for Transcultural Studies in Health, Department of Mental Health and Social Work, Middlesex University UK, The Burroughs, London NW4 4BT, UK; m Professor, Lately at the Department of Nursing, Cyprus University of Technology, 15 Vragadinou str, 3041, Limassol, Cyprus; n Lecturer in Surgical Nursing, Department of Nursing, Cyprus University of Technology, 15 Vragadinou str, 3041, Limassol, Cyprus

**Keywords:** Parenting skills, Migrant/refugee family, Cultural competence, Narratives

## Abstract

Parenting in the context of migration presents a unique set of challenges for refugee parents, who must navigate the cultural norms and expectations of both their home and resettlement countries while balancing their daily parenting responsibilities and practices. This study aims to provide a critical analysis of the experiences, needs, and challenges faced by migrant and refugee parents during their journey and settlement in Europe, as recounted through their personal narratives. Utilizing a qualitative approach, the researchers collected twenty-seven life narratives of migrant and/or refugee parents through purposive sampling. An analysis of the narratives identified four overarching themes that represent the primary challenges faced by refugee families and the need for support. These macro-themes include up rootedness, spatio-temporal uncertainty, trauma and abuses, and parental powerlessness. Parental powerlessness emerged as a synthesis of the causes of trauma from the previous three challenges and highlights the increased loss of parental identity and self-conflict that refugee parents experience. The study reveals that refugee parents face various challenges and barriers, such as language barriers, lack of information and awareness, and cultural differences. It is crucial for healthcare providers and policymakers to consider these findings and develop targeted interventions, such as utilizing interpreters, cultural mediators, and providing culturally sensitive and appropriate healthcare and educational services, as well as implementing specific policies to enhance the health and well-being of refugee parents and their children.

## 1 Introduction

The world is currently experiencing the largest movement of people in human history (Chamie, 2020) with over 1.9 million individuals migrating from crisis countries to Europe in 2020 (International Organization for Migration 2020). Consequently, issues related to migration are at the forefront of public discussions in Europe. According to the United Nation Refugee Agency (UNHCR) in 2019, more than half of the refugees and migrants in Europe and globally were women and children (UNHCR 2019). Refugee families need support to deal with the everyday issues and the long-term effects related to migration to gain a normal life in the host country (Bergset and Ulvik, 2021). As a result, there is an increasing demand for health and social care services among refugees and migrants in Europe (Schilling et al., 2017).

Parenting is a complex and multifaceted process that plays a critical role in a child's developmental outcomes (Pezerović et al., 2019). It involves providing children with the necessary physical, emotional, and cognitive support, as well as creating a nurturing environment that fosters their growth and well-being (Hoghughi, 1998; Chuibin and Fakhra, 2022). However, both mainstream and refugee families face common challenges such as parental and child physical or mental health issues, financial burdens, behavioral difficulties, and social isolation (Lewig et al., 2010). Migration, in particular, can be a significant source of stress for parents due to experiences of torture and trauma, separation from family members, changes in family dynamics and relationships, and concerns for their children (Ayika et al., 2018).

Parenting within the context of migration is particularly challenging (Bergset and Ulvik, 2021) with compound difficulties (Merry et al., 2017) and pressures faced by refugee parents (Riggs J. Yelland et al., 2016). They must find a balance between their culture and the culture of the resettlement country while dealing with everyday responsibilities (Leyendecker et al., 2018). These challenges can have an impact on parenting and lead to increased risks of mental health problems for children (Bryant et al., 2018; Kim et al., 2018). Displaced families face significant challenges that can impede their ability to navigate their new environments and maintain their parenting practices. These obstacles include discrimination, culture shock, language barriers, and employment difficulties, among others (Stewart et al., 2018). Displacement often results in the severance of familial and social connections that provided families with a sense of support, and can leave them without the social structures that typically reinforce their parenting values and practices (Thorleifsson, 2014). As a result, displaced families are forced to confront unique obstacles in their efforts to maintain effective parenting practices, which can have implications for their children's developmental outcomes (Ochocka and Janzen, 2008; Bergnehr, 2019).

The well-being of refugee and migrant children is heavily influenced by the quality of their family environment and parenting practices, as effective parental support plays a crucial role in promoting positive child outcomes (El-Khani et al., 2016). Although refugee parents often express a desire for support from both peers and professionals, their preferences are rarely taken into account when designing support interventions (Stewart et al., 2018). In order to develop effective interventions that meet the needs of refugee families, a deeper understanding of their support requirements (El-Khani et al., 2018) and perspectives on parenting and integration (Bergset and Ulvik, 2021) is necessary.

Refugees in different countries face unique challenges and support systems that significantly impact their mental health and coping strategies. Factors such as the duration of their stay in the current country, the quality of their living conditions, and their access to services all play crucial roles in shaping their mental health and coping mechanisms. This research is the first of its kind to analyze data from refugees across six different European countries, treating them as a unified sample while considering the diverse living conditions they experience. It captures a holistic understanding of refugee experiences across Europe.

Successful integration of refugee and migrant families into host societies can be facilitated by creating supportive environments and strengthening parenting skills through targeted interventions (Fegert et al., 2018).

### Aim

The primary objective of this paper is to provide a critical analysis of the experiences, needs, and challenges faced by migrants and refugee parents during their journey and settlement in Europe, as recounted through their personal stories. These stories were collected as part of the ERASMUS+ project, "Empowering migrant and refugee families with parenting skills" (Intercultural Education of Nurses in Europe - IENE 8 – Project: Grant ID:2018–1-CY01-KA202–046,848).

## 2 Methodology

### 3.1 *Design and sampling*

A qualitative approach was used in this study between January and March 2020, involving twenty-seven (*n* = 27) life narratives from migrant and/or refugee parents, offering a thorough insight into the challenges these families encounter. Narrative inquiry is a valuable technique in qualitative research, enabling the exploration of lived experiences and individual subjectivity through storytelling. This method provides a unique perspective on personal experiences (Eastmond, 2007). The qualitative "life story method" was used to gather these stories, as it offers an approach that delves beyond mere verbal expressions (Ghorashi, 2007) and provides insights into the diverse experiences of forced migration and the meaning of being a 'refugee'. Specifically, this method captures how individuals lived through their experiences ("life as lived"), how they perceived these experiences ("life as experienced"), and how they articulated their experiences in the specific context of their resettlement ("life as told") (Eastmond, 2007).

The narratives were gathered with the help of health and/or social professionals and volunteers who had direct access to and worked closely with migrant/refugee families. Additionally, some participants contributed their own stories directly through a mobile application. Closed questions were included to ensure that all key issues under analysis were addressed by each participant. According to Sowicz et al. (Sowicz et al., 2019) closing questions enhance existing data when analyzed together. Participants were selected through purposive sampling, which involves choosing individuals based on the researchers' judgment of who would provide the most relevant and informative data (Polit and Beck, 2017). We estimated that recruiting 25–30 migrant/refugee families from six European countries would achieve data saturation, considering the time constraints of the research program and the anticipated difficulty of accessing migrant/refugee families at the asylum centers. This resulted in a target sample of 27 families overall. Data saturation was confirmed first by continuously reviewing the analysis and participant quotes to ensure no new significant information was emerging, and second by including closed questions to cover all important topics. This justifies the use of purposive sampling as it effectively captured the nuanced and meaningful experiences of the participants, aligning with recent research that highlights its efficiency over random sampling in qualitative studies (Luborsky and Rubinstein, 1995; Marshall, 1996; Patton, 1990; van Rijnsoever, 2017).

### 3.2 *Tool*

For data collection, we used a semi-structured tool based on two sources: (1) the IENE 8 scoping review of literature on challenges faced by migrant and refugee families with children specifically related to support for parenting skills (C et al., 2023) and (2) the Papadopoulos, Tilki and Taylor Model as adapted for training caregivers of migrant and refugee families in transit (Papadopoulos et al., 2022).

The data collection tool used in this study comprised a series of open-ended questions designed to gather information about the participants' journey, including their country of origin and destination, family status, and the duration of their journey. Participants were also asked to describe their daily living conditions in the country and/or refugee camp where they resided during the data collection period. In addition, the tool included four closed questions focused on parenting challenges, their impact on participants, adaptation to their new life, and the support they found helpful. These questions were included to ensure that all key issues under analysis were addressed by each participant. According to Sowicz, et al. (Sowicz et al., 2019) closing questions enhance existing data when analyzed together.

### 3.3 *Data collection and analysis*

The data collection process for this study involved the use of semi-structured interviews, which were conducted by health and social professionals or volunteers. Participants were also provided with the option to share their stories online through a mobile app specifically developed as part of the IENE 8 project. Interviews were conducted in the language of each IENE 8 partner (DE, EL, EN, IT, RO), and interpreters were used as necessary. All stories were translated into all partner languages, as well as Arabic and Farsi. To analyze the data, thematic analysis was used for qualitative data. The thematic analysis followed the steps outlined by Braun and Clarke (Braun and Clarke, 2006).

### 3.4 *Ethical considerations*

The interviews conducted with refugee parents, required ethical approval only in Greece, where direct interviews were conducted by the Ethics Committee of Doctors of the World/Médecins du Monde - Greece (MdM-Greece), and the study was endorsed by the MdM Board of Directors (Ref. 255). Information sheets and consent forms, written in participants' native languages, were provided to all refugees who agreed to participate. These documents clearly explained the study's purpose, ensuring that participation was entirely voluntary and anonymous. Participants were informed that they could refuse or withdraw from the study at any time without penalty or loss of benefits. This included assurance that their access to services or benefits would not be affected by their decision. Informed consent was obtained through signed forms, which participants reviewed in their native language, with opportunities to ask questions before agreeing to take part. To maintain data confidentiality, personal identifiers were removed or anonymized, and all data were securely stored with access restricted to the research team.

## 4 Findings

The journey of resettlement for migrant and refugee families is fraught with numerous challenges, as reflected through their personal storytelling narratives.

### 4.1 *Demographic and social characteristics*

Our findings highlight a variety of family structures among the participants. More than half of the families (56 %) were identified as nuclear families, 37 % as single-parent households, and 7 % as extended families. This diversity highlighting the complexities many parents encountered in providing and caring for their children during migration. These families were residing across several European countries, including Greece, the UK, Germany, Cyprus, Italy, and Romania. A significant proportion of the families (56 %) originated from Syria, with others coming from countries like Afghanistan, Iraq, Nigeria, and Iran.

Most of the families spent between a few months (1–5 months, 38.5 %) and up to four years in transit before reaching their final destination. For the majority, the journey as refugees lasted between 1 and 2 years (57.7 %). The length of stay in their host countries varied significantly, ranging from a few months (34.6 %) to several years. Most families had been living in their host country for 2 to 5 years (53.84 %). By the time of data collection, three families (11,53 %) had been residing in the host country for over 10 years.

### 4.2 *Personal narratives*

The narratives collected depict the day-to-day struggles of parents during their migration to Europe. The analysis identified four macro-themes: **uprootedness, spatio-temporal uncertainty, trauma and abuse**, and **parental powerlessness**. These themes illustrate the multifaceted challenges refugee parents face, particularly the erosion of parental identity and the internal conflict many experiences.

#### 4.2.1 Theme 1: uprootedness

***Subtheme 1: Living Situation in the Home Country Before Departure***

Many parents described being compelled to leave their home countries due to safety concerns and the desire for a better future and education for their children. For instance, one parent recounted:

"We lost many family members in a few days. The fear of our imminent death or the death of our children made us want to leave… What can we forget from the war… the bombs?" (Story 3)

“I am a father of two children, I had a very good job in Syria in a company which was bombed as well as our home due to shootings. Our two children, aged 10 and 12 years old, saw their grandfather, namely my father, being injured and dying in front of their eyes. We were forced to leave in order to save our lives.” (Story 2)

Another father shared the traumatic experience of his children witnessing their grandfather's death:

"Our two children, aged 10 and 12 years old, saw their grandfather, namely my father, being injured and dying in front of their eyes. We were forced to leave in order to save our lives." (Story 2)

***Subtheme 2: The Difficult Journey***

The migration journey has profound and lasting psychological, emotional, and physical effects on individuals. Our data reveal that the migration experience is often complex and fraught with danger, with many migrants enduring extended and perilous journeys. Participants reported navigating non-linear routes through multiple countries before arriving at their final destination. This extended and challenging journey significantly impacts their well-being and highlights the multifaceted nature of their migration experiences.

"From Syria to Turkey, from there by boat to Greece alone with my children. From Greece by train to Macedonia, Serbia, Croatia, Slovenia, Germany." (Story 27)

“The escape to Germany was difficult. We were in a boat with 30 people. It was raining and the waves were high. Then the engine has failed. A big ship was nearby, but they did not help us. Everyone was very scared. I told the kids to close their eyes”. (Story 23)

One mother recalled a particularly harrowing moment:

"While crossing the river, I had to carry the baby, which fell into the water. I thought for a few seconds that the baby died, and I remember that as the most difficult part of my journey." (Story 4)

#### 4.2.2 Theme 2: Spatio-temporal uncertainty

***Subtheme 1: Run for a Better Life***

The current data reveals that families migrating to new countries often encounter significant challenges upon arrival, despite their initial hope for a better future. These challenges demand substantial resilience and inner strength as families work to overcome obstacles and maintain hope.

“The oldest son, after witnessing a bomb explosion and loss of her grandparents, is facing problems we could not deal with in my country, so we decided to move to a better place”. (Story 6)

"We would like to mention that our children have not fully recovered yet. We really care for the future of those children; they have dreams for their studies and for a better life." (Story 3)

“I try to make my children feel safe and happy. Sometimes I cry. I spend my whole day, taking them to play and learn ……..I try to be patient and hope for the best”. (Story 14)

“The situation improved……but we now have to deal with our health problems. My son and I talked to a psychologist for three months in order to help us face our anxiety and hopelessness. I am worried about my children's future and their education. Will they adapt to their new life?” (Story 15)

“Thanks to this welcome center, I coped with escaping from this nightmare step by step. I found a new and respectable job, and my daughter lives with me, now”. (Story 18)

***Subtheme 2: Adapting to a New Life***

Insights from the current data indicate that refugee families frequently face significant difficulties in establishing new lives and adapting to their host countries. The majority of participants (62.9 %) indicated that they were seeking better living conditions, with 55.5 % aiming to access fair and efficient asylum procedures, and 51.8 % wanting to find a place where they could feel safe. The transition from their previous lives requires a substantial period of adjustment. Their hopes for a better future are heavily dependent on achieving a sense of safety and security, which is crucial for them to live without fear.

"In Germany, I live without fear. I live free. Not in Iraq. Here I am not asked about religion. We can speak Arabic… My son goes to kindergarten, my other to elementary school. My wife is learning German now." (Story 23)

Additionally, the findings reveal that refugees frequently encounter significant disappointment when resettling in their host countries. Participants commonly describe their experiences of living in overcrowded and substandard conditions, which complicates their efforts to rebuild their lives. Furthermore, challenges in finding employment intensify these difficulties, hindering their pursuit of stability and successful integration. One mother from Greece described her dire situation:

“The daily living conditions are inhumane for me and for my family. We were living in a squat in Exarchia (an area in the centre of Athens). Suddenly they obliged us to leave from there and now we are living in a tent just outside of a refugee camp. I am really afraid for the health condition of my children, because one of my daughters suffers from a disease in the kidneys. Also, due to the fact that we are staying in a tent and it is really cold, I am afraid for frostbites” (Story 7)

“When we reached Greece, I felt safe for a while, but the camp was really crowded and people from all over the world strived for their survival. There were a lot of conflicts. The situation improved in Athens but we now have to deal with our health problems.” (Story 15)

Additionally, challenges in finding employment intensify these difficulties, hindering their pursuit of stability and successful integration, as highlighted by one single father:

"*Difficult to find a job as a foreigner. I am a single parent (father), and I can't support my child…"* (Story 21)

“In Italy, I can't find a job just my family reach me. I work irregularly. I mind them, but, really, I feel powerless.” (Story 17)

“I am living in a country where I do not speak the language, my husband tries a lot to find a job, but this is difficult….. I do not know what to do”(Story 6)

*“How am I supposed to learn the language or get a job when I don't have time even to take a bath?”* (Story 8)

***Subtheme 3: Education***

In their narratives, participants mentioned that their primary motivation for migration is to secure better educational opportunities and a safer living environment for their children. A substantial 44.4 % of families reported that while they wanted their children to have access to education, they faced numerus challenges in ensuring regular school attendance. This frustration was highlighted in two parent's stories:

"Our future was unknown, and we were unable to respond to their questions regarding our future, their education… We really care for the future of our children; they have dreams for their studies and for a better life." (Story 3)

*“My two boys had school problems and language difficulties and also problems with ghetto formation”* (Story 27)

#### 4.2.3 Theme 3: trauma and abuse

***Subtheme 1: Fear***

The majority of participants reported experiencing a range of adverse emotions, including anxiety, stress, worry, fear, sadness, and hopelessness. The sense of fear, as recounted in many of the refugee stories, began before their journey and persisted throughout their travels and during their time in the refugee camps. A substantial proportion of parents (70.3 %) reported feeling helpless and unable to protect their children during the journey, while 44,4 % mentioned that they faced risks of harm and/or violence.

*“Our two children, aged 10 and 12 years old, saw their grandfather, namely my father, being injured and dying in front of their eyes”.* (Story 2)

“I escaped from my country just because a terrible dictatorship ….. some people hit my head with a weapon and I have been close to die. There no law, no rights. Only violence….. I witnessed many rapes against women …..”. (Story 16)

Parents also recounted their traumatic experiences during their time in the refugee camps.

"*Every night, while sleeping, he was ‘seeing’ again and again the moment of the shooting of his grandfather.*" (Story 2)

Parents also reflected on the fear that gripped them during their journey:

"*During the journey, we faced sickness, which led to fear of losing one child.*" (Story 5)

*“During our long journey …… there were a lot of times I was afraid for my life and my children's safety”.* (Story 13)

***Subtheme 2: Separated Families***

Many of the migrants and refugees who participated in this study (18,5 %) reported leaving their families behind in pursuit of safety and employment opportunities. Similarly, the data revealed that numerous migrant children were separated from their parents for the same reasons, with the intention of being reunited after resettlement. The findings also indicate that, such separation can have a significant impact on the emotional and social development of children. Separation of family members in the context of displacement emerged as a prevalent occurrence in the study.

"At the moment, I am paying for a small room to live with my two young sons, as the three daughters left illegally to Switzerland. I am now looking forward to reunite with them soon." (Story 5)

“The most difficult part of my journey was the fact that I had firstly to deal with the fact that my daughter did not manage to leave Turkey with the rest of the family, as she was arrested by the Police”. (Story 1)

“I had to leave my son behind as he was not accepted by the Asylum Service in order to reunify with the rest of the family in the Netherlands.” (Story 1)

The longing for reunification underscored the ongoing struggles faced by refugee families, with many expressing a desire for better living conditions (62,9 %), fair asylum procedures (55,5 %), and a safe environment (51,8 %).

#### 4.2.4 Theme 4: Parental powerlessness

Parental powerlessness emerges as a synthesis of the traumatic experiences and challenges faced by refugee families across the three themes of uprootedness, spatio-temporal uncertainty, and trauma and abuse. Feelings of powerlessness were prevalent among the parents in this study, exacerbated by the overwhelming challenges of migration and resettlement. Many parents employed various coping mechanisms to deal with their difficulties. A majority (62.9 %) discussed their family's trauma with healthcare professionals, while 59.2 % sought support from volunteers and workers. The strength derived from professional mental health support (reported by 55.5 %) provided some respite amid their challenges. Many of the stories capture the essence of parental powerlessness:

"In Italy, I can't find a job just my family reach me. I work irregularly. I mind them, but, really, I feel powerless." (Story 17)

“We were really worried for our children, as they were having sleeping difficulties and nightmares, due to the fact that many friends and close relatives had recently died. Our future was unknown and we were unable to respond to their questions regarding our future, their education or whether we would return back to our home, to their friends, to their school..” (Story 3)

“I feel exhausted as I cannot sleep at nights. I should look after two other children who need their mother and I am afraid I will explode. I am living in a country where I do not speak the language, my husband tries a lot to find a job, but I also need him in our home, I do not know what to do”. (Story 6).

*“How am I supposed to learn the language or get a job when I don't have time even to take a bath?”* (Story 8)

“Since the beginning of my difficult journey from Iran, I am in a very bad psychological situation, I feel tired and desperate for the psychosomatic suffering of my children, as many times we need to cover big distances by foot”. (Story 9)

“I'm terrified my wife and children are still alone… in Italy, I can't find a job just my family reach me. I work irregularly. I mind them, but, really, I feel powerless”. (Story 17)

## 3 Discussion

The findings of this study highlight the complex challenges that refugee families face when they arrive in a new country. The results show how deeply uprootedness, uncertainty, and trauma affect these families, especially in their roles as parents.

***Uprootedness: Challenges of Leaving the Home Country***

The theme of uprootedness reflects the deep disruption in the lives of refugee families forced to leave their home countries for safety reasons. As stated by the United Nations High Commissioner for Refugees (UNHCR), "No one chooses to become a refugee" (Martin, 2021). This profound truth underscores the context of their experiences: individuals who have survived war, famine, and instability, and now seek a more secure environment for themselves and their children (International Organization for Migration 2018). Many parents in our study shared the extreme situations that made them flee, often involving life-threatening dangers. This aligns with existing research on the severe stress and trauma linked to forced migration due to war or persecution (Masten and Cicchetti, 2016; A. d'Abreu et al., 2021). The stories in our study, including those about the traumatic loss of loved ones, highlight the significant psychological impact of these experiences.

The migration journey was described as dangerous and difficult, often involving travel through several countries in complicated ways. The physical and emotional strain of this journey is clear from the participants' stories. Long periods of danger, uncertainty, and harsh conditions have left deep impacts on these individuals. For example, one mother almost lost her baby while crossing a river, showing the life-threatening risks they encountered. These stories match previous research that highlights the extreme challenges forced migrants face during their journeys (Merry et al., 2017). Despite the significant risk of death and harm during the journey, the prospect of survival and the promise of a better future outweighed these dangers.

Family separation was another major issue. Many participants were separated from family members during their journey, which had significant emotional impacts on both parents and children (UNHCR 1951; Bentley Waddoups and H.Yoshikawa, 2019; Amnesty International UK 2019). The difficulties in reuniting with family, often due to bureaucratic and legal hurdles, highlight the need for better family reunification processes (Milios, 2021; Boreil et al., 2020). The Final Act of the UN Conference on the Status of Refugees and Stateless Persons (1951) emphasizes the importance of family unity, stating it is a fundamental right of refugees (UNHCR 1951). Improving family reunification efforts is essential to support refugee families and reduce the trauma of separation.

***Spatio-Temporal Uncertainty: Navigating a New Life***

Refugee families face major challenges when adapting to life in a new country, marked by spatio-temporal uncertainty. Despite their hopes for a better future, many struggles with finding stable housing, accessing education, and securing jobs. For example, only 44.4 % of parents reported in this study that their children could attend school regularly, showing how hard it is to get consistent educational opportunities. A finding that aligns with previous studies highlighting the educational disruptions faced by refugee children (Dryden-Peterson, 2016). Parents in this study expressed deep concern about their children's future and success in school. The study further underscores the significant impact of displacement on refugee children's education, showing that frequent relocations and the trauma of forced migration often lead to major setbacks in their academic performance and school engagement.

Moreover, recent research has extensively explored the living conditions of refugees, particularly in camps that are intended to provide temporary shelter and aid (Ramadan, 2013) but often become sites of prolonged insecurity and violence (Lalla et al., 2020; Turner, 2020). Substandard living conditions, combined with inadequate access to healthcare, frequently result in poor health outcomes for refugees, as documented by studies on refugee health disparities (Jesuit Social Services 2015; Mendola and Busetta, 2018; Fazel et al., 2020). These environmental stressors further complicate the resettlement process, intensifying the spatio-temporal uncertainty that refugees experience.

Employment is another critical challenge, that some participants described difficulties of finding stable jobs due to language barriers and their foreign status, which is consistent with the literature on refugee integration (Ager and Strang, 2008). Additionally, despite having good jobs in their home country, participants struggled to find similar or well-paying positions in the host country. This issue is highlighted in the report *"I Hate Being Idle",* which notes that even highly skilled and well-educated refugees frequently end up in roles that do not match their level of expertise in their new country of resettlement (Doyle, 2019). These employment challenges contribute to increased stress and uncertainty, making it harder for refugees to establish a stable and normal life in their new environment. The literature also suggests that unemployment and underemployment are common among refugees, leading to prolonged economic instability and social exclusion (Phillips, 2017).

Although only 44.4 % of respondents mentioned language as a primary issue, this likely reflects broader difficulties with integration. Language barriers not only affect communication but also limit access to essential services, including health care and education for children (Jou, 2012; Bradley et al., 2017). Additionally, other research has shown that proficiency in the local language is key to successful integration and reducing acculturative stress (McCleary, 2017; Raghallaigh et al., 2021). The fact that only 44.4 % of respondents mentioned both language and their children's education suggest that other, more immediate concerns like safety and basic needs may have been more pressing during their initial adjustment period (S. Merry et al., 2017). This is further supported by participants' stories in this study, where ‘better living conditions’ (62.9 %), ‘access to fair and efficient asylum procedures’ (55.5 %) and feelings of safety (51.8 %) were cited as the most important needs and forms of support. Betancourt et al. (Betancourt et al., 2015) also note that the stress and trauma of forced migration can cause families to focus on securing basic needs before addressing challenges like learning a new language or ensuring their children's education.

***Trauma and Abuse: The Enduring Impact of Fear***

The theme of trauma and abuse emerged strongly in the data, with many participants reporting feelings of fear, anxiety, and helplessness. These emotions were not only tied to their experiences before and during their journey but also persisted after their arrival in the host country. The narratives collected in our study vividly illustrate the ongoing psychological impact of these traumatic experiences, such as children reliving the traumatic death of a grandparent or parents fearing for their children's safety during the journey. The pressure to adapt to a new environment places a significant strain on refugees and their relationships (Bellinger, 2013), leading to feelings of loneliness, rejection, and isolation (Chen and Renzah, 2017), as supported by findings in refugee mental health literature (Kirmayer et al., 2011; Kirmayer et al., 2019).

The cumulative challenges of trauma, negotiating new social roles, managing socioeconomic disadvantages (Mude and Mwanri, 2020), and navigating cultural differences are well-documented in the literature (Masten and Cicchetti, 2016). These issues often result in difficulties managing school, unemployment, and language barriers, which are critical factors influencing the success of refugee integration (Mitchell and Kamenarac, 2021; Jacolyn et al., 2021; Colic-Peisker and Walker, 2003; Stewart et al., 2018).

Interestingly, findings from this study show that 70.3 % of parents felt helpless in protecting their children, the qualitative narratives provide a deeper understanding of this helplessness, linking it to specific traumatic events and the ongoing stress of adapting to a new environment. This aligns with previous research (Merry et al., 2017) that emphasizes the importance of parental resilience in navigating the challenges of resettlement. Moreover, the literature suggests that the responsibilities of parenthood can be a significant source of strength for forced migrants, helping them overcome the hardships they face. Parenthood not only enhances parental resilience but also plays a crucial role in fostering resilience in children, highlighting the interconnectedness of family dynamics in the context of forced migration (Masten and Cicchetti, 2016; Masten and Palmer, 2019).

***Parental Powerlessness: Erosion of Control and Identity***

The combined impact of uprootedness, uncertainty, and trauma often leads to a deep sense of parental powerlessness, as many participants in our study described. This feeling of powerlessness involves a loss of control and authority, internal conflict, and a weakening of parental identity. Parents expressed being overwhelmed by their inability to meet their children's needs, which was further intensified by financial insecurity and cultural displacement.

One parent highlighted the struggle of balancing the demands of finding work while caring for their children, illustrating the internal conflict and self-doubt many parents face. This theme is consistent with existing research on the challenges of maintaining parental identity and authority in forced migration contexts (Masten and Palmer, 2019).

***Positive Reframing and Resilience***

Despite the many challenges, some parents in our study managed to stay positive and use their coping skills effectively. They found that building connections through community, family, and friends was crucial for adapting to their new life. This finding is consistent with existing literature on the importance of social support in fostering resilience among refugees (Ager and Strang, 2008; A. d'Abreu et al., 2021).

Participants reported specific difficulties, such as teaching their children cultural stories and songs, which added to their stress. To manage these challenges, families implemented strategies to preserve their cultural identity. For instance, one participant shared that, *"I am teaching my children stories and songs from our culture,"* highlighting the struggle to balance their cultural values with those of the host country, especially in raising their children. This approach aligns with previous research, which underscores the importance of maintaining cultural practices in supporting the well-being and identity of refugee families (Weine, 2008).

Moreover, the participants expressed gratitude for finding safety and held onto hope for a better future. This positive reframing appears to enhance their resilience and determination to build a new life for their families. This supports research highlighting the impact of positive reframing on resilience among forced migrants (A. d'Abreu et al., 2021).

Effective mental health care requires health professionals to possess strong communication skills and cultural competence (Mechili et al., 2018). The necessity of cultural expertise and interdisciplinary collaboration is further emphasized by Dubus' study (Dubus, 2021), which suggests that regular professional meetings can improve support for refugee families.

This study adds to the existing literature by offering a deeper understanding of the specific challenges refugee parents face and the coping strategies they use.

## 4 Limitations

It is important to acknowledge the limitations of this study. Firstly, selection bias is a possibility in this narrative-based research. The group of refugee parents who participated in this study may have distinct characteristics that led them to flee to their respective host countries and come into contact with health professionals or volunteers who were involved in collecting the data. Therefore, caution must be exercised when generalizing the findings to other populations. To enhance the transferability, dependability and trustworthiness of future studies on migrant parents' lived experiences, it is recommended that a range of research methodologies be employed. This would help to bolster the body of knowledge on this topic, which could then inform the development of targeted interventions and policy decisions.

A limitation of this qualitative research is the diversity of conditions in the different European countries where the refugee families were hosted. These varying conditions—ranging from levels of support to challenges in integration and employment—affect the experiences and perspectives of refugee families. While this research offers valuable insights, the diversity of host country conditions highlights the need for careful consideration when interpreting the findings and underscores the importance of context-specific policies and interventions.

Another limitation of this study lies in the potential impact of the researchers' own perspectives, biases, and preconceptions on the analysis and interpretation of the data. As qualitative research often involves a degree of subjectivity, the researchers' own experiences, cultural backgrounds, and beliefs may have influenced the way they interpreted the narratives shared by the refugee parents. While efforts were made to remain objective and reflexive throughout the research process, it is important to acknowledge that complete neutrality is difficult to achieve. These factors may have subtly shaped the themes that emerged from the data, potentially affecting the study's conclusions. Future research could benefit from incorporating multiple analysts with diverse backgrounds to mitigate the influence of individual biases and enhance the robustness of the findings.

## 5 Conclusion

This study sheds light on the experiences of refugee parents in resettlement countries, specifically in accessing healthcare services and education for their children, as well as a safe home. The findings highlight the challenges and barriers that these parents face, including language barriers, lack of information and awareness, and cultural differences. These barriers can have negative consequences for the health and well-being of their children. It is crucial for healthcare providers and policymakers to consider these findings and develop targeted interventions, such as utilizing interpreters, cultural mediators, and providing culturally sensitive and appropriate healthcare and educational services, as well as implementing specific policies to enhance the health and well-being of refugee parents and their children..

In order to build on the findings of this study, future research should explore a broader range of refugee populations and contexts to enhance our understanding of the diverse experiences of refugee parents in different resettlement environments. Longitudinal studies could provide valuable insights into how these challenges evolve over time and the long-term impact on children's health and well-being. Additionally, research could focus on evaluating the effectiveness of specific interventions, such as the use of interpreters and cultural mediators, to determine best practices for supporting refugee families. Exploring the role of social networks, community support systems, and the impact of policy changes on refugee parents' access to healthcare and education could also provide valuable information to inform future interventions and policies.

## CRediT authorship contribution statement

**Elena Rousou:** Conceptualization, Methodology, Writing – original draft, Writing – review & editing. **Paraskevi Apostolara:** Conceptualization, Methodology, Writing – original draft, Writing – review & editing. **Venetia Sofia Velonaki:** Writing – review & editing. **Irena Papadopoulos:** Conceptualization, Methodology, Writing – review & editing. **Athena Kalokairinou:** Methodology. **Ourania Sakellaraki:** Methodology. **Victor Dudau:** Methodology. **Andrea Kuckert:** Methodology. **Runa Lazzarino:** Methodology, Resources. **Manuela Mauceri:** Methodology. **Alfonso Pezzella:** Methodology, Resources. **Christiana Kouta:** Conceptualization, Methodology, Writing – original draft. **Theologia Tsitsi:** Writing – review & editing.

## Declaration of competing interest

The authors declare that they have no known competing financial interests or personal relationships that could have appeared to influence the work reported in this paper.
